# Integrated transcriptomic and metabolomic analyses reveal critical gene regulatory network in response to drought stress in *Dendrobium nobile* Lindl

**DOI:** 10.1186/s12870-025-06176-8

**Published:** 2025-02-04

**Authors:** Chaoyan Lv, Ya He, Zaiqian Jiang, Wenjia Hu, Mei Zhang

**Affiliations:** College of Biology and Agriculture, Zunyi Normal University, Zunyi, 563006 Guizhou China

**Keywords:** *Dendrobium nobile* Lindl, Drought Stress, Transcriptome, Metabolome, Regulatory Network

## Abstract

**Background:**

*Dendrobium nobile* Lindl belongs to the genus *Dendrobium* of the orchid family and is a valuable herbal medicine. Drought stress severely affects the growth of *D. nobile* Lindl; however, the specific regulatory mechanisms have not yet been elucidated.

**Results:**

In the present study, we conducted a combined transcriptome and metabolome analysis of *D. nobile* Lindl stems under different drought stress conditions. Global transcriptomic changes were detected in *Dendrobium* under different drought stress conditions. KEGG enrichment analysis showed that the DEGs were enriched in plant hormone signal transduction; cutin, suberin, and wax biosynthesis; starch and sucrose metabolism; and the biosynthesis of various plant secondary metabolites. The differentially abundant metabolites (DAMs) detected using STEM analysis were enriched in pathways associated with glucosinolate biosynthesis and cyanoamino acid metabolism. We constructed a regulatory network for the drought tolerance of *Dendrobium* by weighted gene co-expression analysis.

**Conclusions:**

The results showed that arginine and proline metabolism, glucosinolate biosynthesis and tyrosine metabolism pathways participated in regulating drought stress in *D. nobile* Lindl. Our study provides a theoretical basis for studying the drought resistance mechanisms in *Dendrobium*.

**Supplementary Information:**

The online version contains supplementary material available at 10.1186/s12870-025-06176-8.

## Background

*Dendrobium nobile* Lindl. is a plant of the genus *Dendrobium* in the Orchidaceae family [1]. It was the earliest identified species of *Dendrobium* among ancient Chinese medicinal herbs and is one of the main varieties of medicinal *Dendrobiums* in China [2]. *D. nobile* Lindl is an important medicinal plant that benefits the stomach, generates fluids, brightens the eyes, strengthens the body, moistens the lungs, and relieves cough [3]. Its flower color, posture, and shape are beautiful and display ornamental value. Variants of *D. nobile* Lindl from different geographic regions may be numerous, and researchers have developed specific sequence-characterized amplified region (SCAR) markers for the identification of specific variants of this plant [4]. *Dendrobium* is an epiphytic orchid that prefers a semi-shaded environment and warm and humid climate, mostly epiphytic in the branches of trees or rock crevices under the forest at an altitude of 500–1,800 m [5]. However, the epiphytic nature causes the root system to be exposed to air for a long time, and it cannot obtain water from the soil like land plants. Its water sources are poor and difficult to obtain. Consequently, *Dendrobium* often suffers from short-or long-term drought stress [6]. Currently, there are more studies on drought stress in *D. officinale* and fewer related studies in *D. nobile.* Existing studies have found that ALDH18A was involved in the TCA cycle of plant, affects amino acid metabolism, which may play a regulatory function in counteracting drought environments [7]. Rocf encodes arginase that is involved in urea synthesis and is important for plants to cope with water stress [8]. Studies have shown that P4HA, a key gene for amino acid metabolism, was involved in drought resistance in plants [9]. Researchers have discovered a relationship between the codon usage preference of antimicrobial peptides and drought stress in *D. officinale* [10]. The ectopic expression of DoFLS1 in *D. officinale* may enhance drought stress tolerance [11].

Drought conditions reduce total *Dendrobium* accumulation, which affects *D. nobile* Lindl production [12]. *DNMSI1* is a negative regulatory factor in drought-stress of *D. nobile* Lindl [13]. However, the regulatory network of the drought stress response in *D. nobile* Lindl has not yet been elucidated. In this study, we performed transcriptomic and metabolomic analyses of *D. nobile* Lindl under different water stress conditions. Arginine and proline metabolism, glucosinolate biosynthesis, and tyrosine metabolism pathways participated in the regulation of drought stress in *Dendrobium nobile* Lindl. Within the arginine and proline metabolic pathways, genes such as ALDH18A, rocF, proC, P4HA, arginine decarboxylase, and speE showed an increasing trend during drought stress. In the glucosinolate biosynthesis pathway, MAM1 and metabolites such as 2-oxo-3-methylthio-butanoic acid, 2-oxo-4-methylthio-pentanoic acid, and l-leucine, were upregulated. Genes in the tyrosine metabolism pathway, including polyphenol oxidase, PAT, AOC3, maiA, and FAH showed an increasing trend. Our study provides a theoretical basis for studying the drought resistance mechanisms in *Dendrobium*.

## Results

### Characterization of different drought stress conditions in *Dendrobium*

**Fig. 1:**
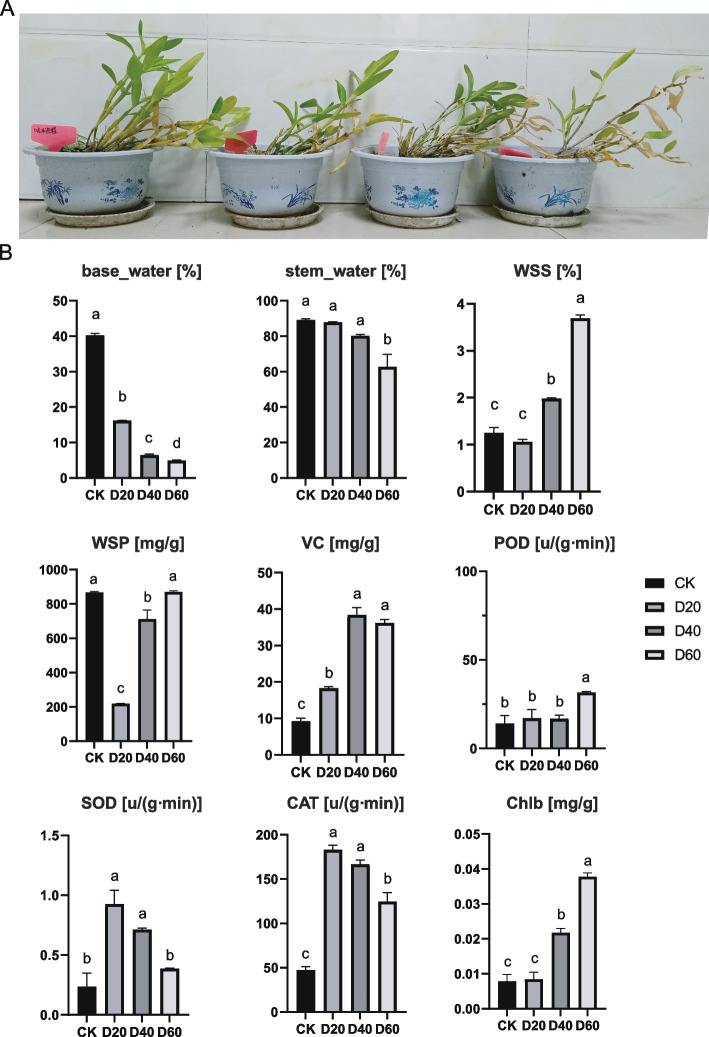
Changes of *Dendrobium* under different drought stress conditions: morphological changes (**A**) and physiological properties such as soil substrate moisture content (base_water), stem moisture content (stem_water), and the levels of soluble protein (WSP), soluble sugar (WSS), ascorbic acid (VC), superoxide dismutase (SOD), peroxidase (POD), catalase (CAT), and chlorophyll B (ChlB) in *Dendrobium* samples (**B**)

Significant morphological changes were observed in *D. nobile*. In the control group (CK), plants exhibited overall health, with green leaves and robust growth (Fig. 1A). The leaves of the D20 plants showed slight yellowing and fatigue during growth (Fig. 1A). In contrast, the D40 plants exhibited pronounced leaf yellowing, with some leaves wilting and falling off (Fig. 1A). Growth was noticeably inhibited, and the stems appeared wilted (Fig. 1A). By D60, extensive leaf yellowing, and substantial leaf wilting and shedding were observed, indicating severe damage to plant growth (Fig. 1A). Overall, as the drought treatment duration increased, *Dendrobium* leaves gradually transitioned from green to yellow, accompanied by significant growth inhibition, culminating in severe wilting and leaf loss after 60 days of drought.

As the duration of drought stress increased, both the soil substrate water content and stem water content of the plants progressively decreased (Fig. 1B). With the progression of drought treatment, base_water gradually decreased, whereas _water significantly decreased only at D60 (Fig. 1B). Both WSS and Chlb showed gradual increases starting from D40, whereas WSP initially decreased significantly at D20 before gradually returning to normal levels (Fig. 1B). VC also gradually increased, reaching its highest levels at D40 and D60 (Fig. 1B). POD levels increased at D60, whereas SOD and CAT levels increased significantly at D20 and D40, followed by a decrease at D60 (Fig. 1B).

### Global transcriptomic changes in different drought stress conditions of *Dendrobium*

**Fig. 2:**
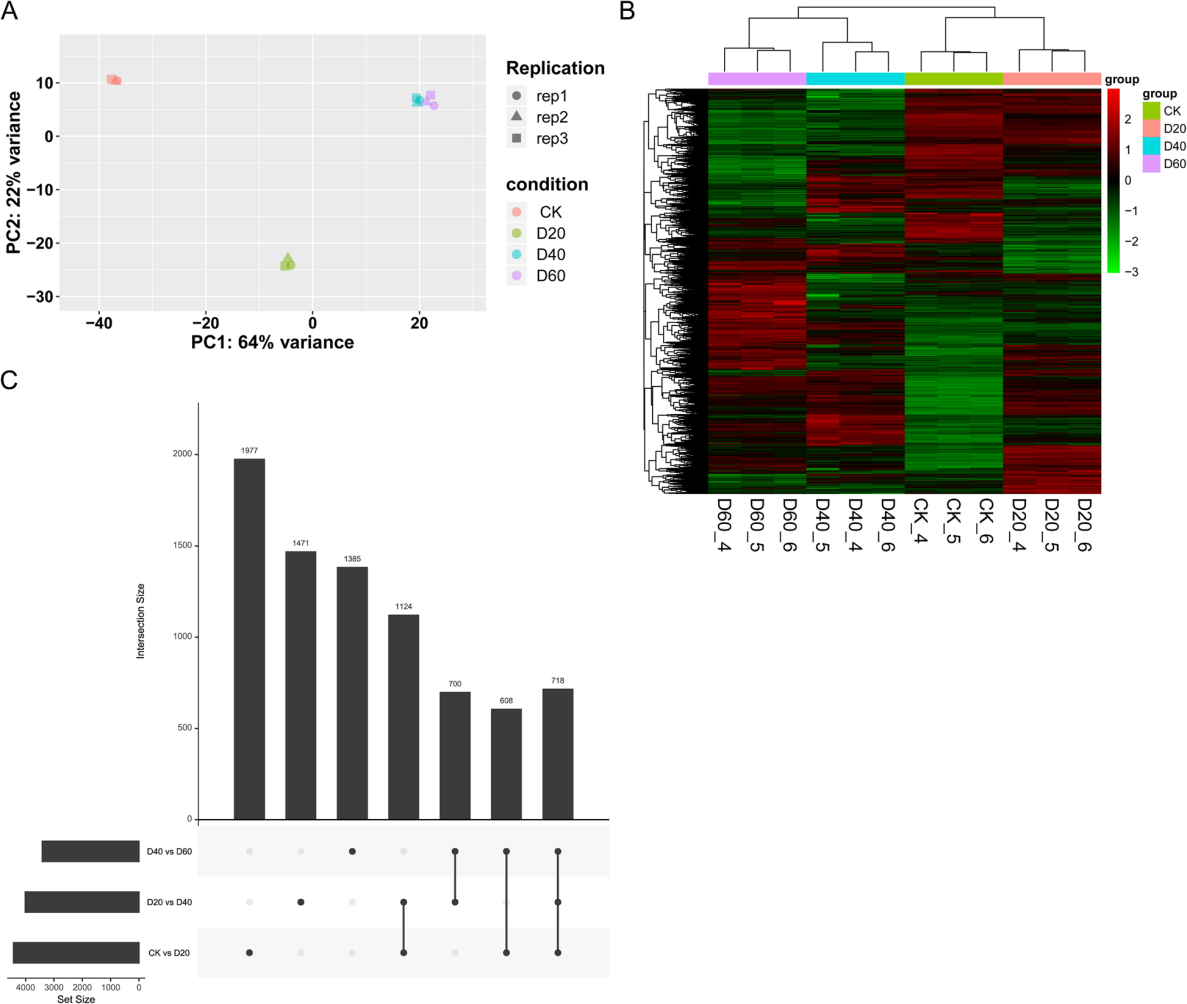
Transcriptomic changes between different drought stress conditions. **A** PCA scatter plot; colors denote different drought stress conditions and shapes represent biological replicates. **B** Heatmap of all DEGs from pairwise comparisons between time points. **C** Venn diagram depicting DEGs from comparisons between adjacent time points (CK vs. D20, D20 vs. D40, D40 vs. D60)

Transcriptome sequencing yielded an average of 13.1 million clean reads per sample after quality control, with an average mapping rate of 86.3% (Supplementary Table S1). Principal component analysis (PCA) indicated that the samples within each group tended to cluster together, suggesting good intragroup consistency (Fig. 2A and Table S2). Samples between groups were widely separated, implying significant differences in the drought resistance levels across different time points in *Dendrobium* (Fig. 2A). Specifically, samples from the D40 and D60 groups were closer than those from the other groups (Fig. 2A).

Differential expression analysis identified the highest number of differentially expressed genes between the CK and D60 groups, followed by those between the CK and D40 groups (Table S3). The number of differentially expressed genes changed gradually with prolonged drought stress (Fig. 2B). The comparisons between adjacent time points (CK vs. D20, D20 vs. D40, and D40 vs. D60) revealed 718 common DEGs (Fig. 2C and Table S4).

### Metabolomic changes associated with different drought stress conditions of *Dendrobium*

**Fig. 3:**
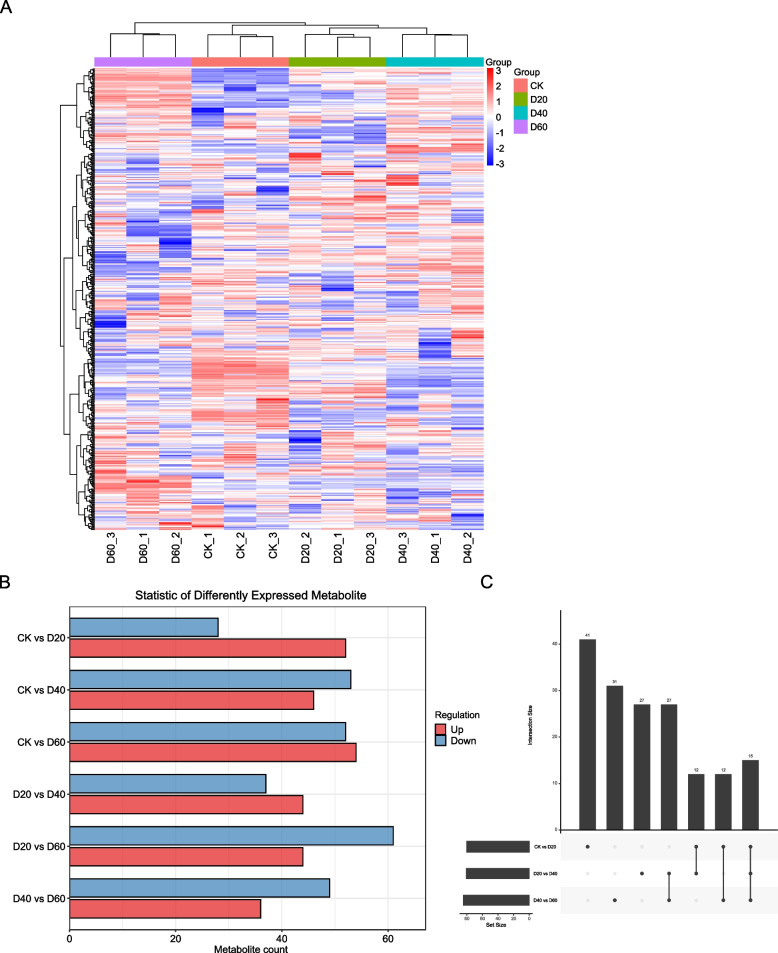
Metabolomic changes between different drought stress conditions. **A** Heatmap of all metabolites. **B** Barplot showing the number of all differentially expressed metabolites from the pairwise comparisons between time points. **C** Venn diagram depicting differentially expressed metabolites from the comparisons between adjacent time points (CK vs. D20, D20 vs. D40, D40 vs. D60)

A total of 475 metabolites were identified based on secondary mass spectrometry (Table S5 and Fig. 3A). The highest number of differentially abundant metabolites (DAMs) was observed between CK and D60 (106) and between D20 and D60 (105) (Fig. 3B, Table S6). Comparisons between adjacent time points (CK vs. D20, D20 vs. D40, and D40 vs. D60) revealed 15 common DAMs (Fig. 3C and Table S7).

### STEM analysis in different drought stress conditions of *Dendrobium*

**Fig. 4:**
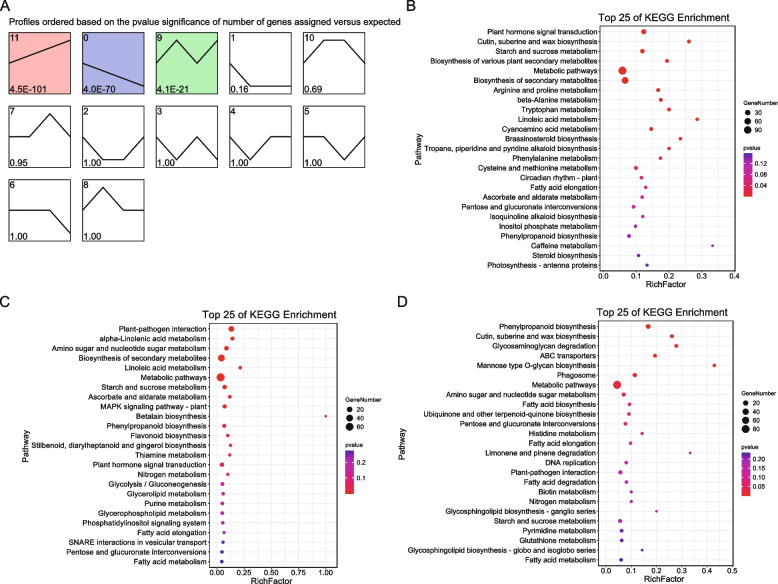
Expression trends of DEGs identified in STEM analysis. **A** Gene profiles identified in STEM, with profiles 11, 0, and 9 highlighted as significant with specific background colors. The numerals in the lower left corner denote the *P-value* of the number of assigned genes versus expected. **B**-**D** KEGG enrichment analysis of genes from profile 11 (**B**), profile 0 (**C**), and profile 9 (**D**)

To explore the temporal trends in gene expression changes under prolonged drought stress, trend analysis using STEM was performed on the DEGs from the transcriptome data. Profiles 11, 9, and 0 were also significantly enriched (Fig. 4A). KEGG enrichment analysis of the genes in profile 11 indicated significant enrichment in pathways such as plant hormone signal transduction; cutin, suberin, and wax biosynthesis; starch and sucrose metabolism; biosynthesis of various plant secondary metabolites; arginine and proline metabolism; beta-alanine metabolism; tryptophan metabolism; linoleic acid metabolism; cyanoamino acid metabolism; brassinosteroid biosynthesis; tropane, piperidine, and pyridine; alkaloid biosynthesis; phenylalanine metabolism; and cysteine and methionine metabolism (Fig. 4B). The genes in profile 0 were mainly enriched in pathways including plant-pathogen interaction, alpha-linolenic acid metabolism, amino sugar and nucleotide sugar metabolism, biosynthesis of secondary metabolites, linoleic acid metabolism, metabolic pathways, starch and sucrose metabolism, ascorbate and aldarate metabolism, MAPK signaling pathway—plant, betalain biosynthesis, phenylpropanoid biosynthesis, and flavonoid biosynthesis (Fig. 4C). Profile 9 genes were enriched in phenylpropanoid biosynthesis; cutin, suberin, and wax biosynthesis; glycosaminoglycan degradation; ABC transporters; mannose type O-glycan biosynthesis; phagosome; and metabolic pathways (Fig. 4D).

**Fig. 5:**
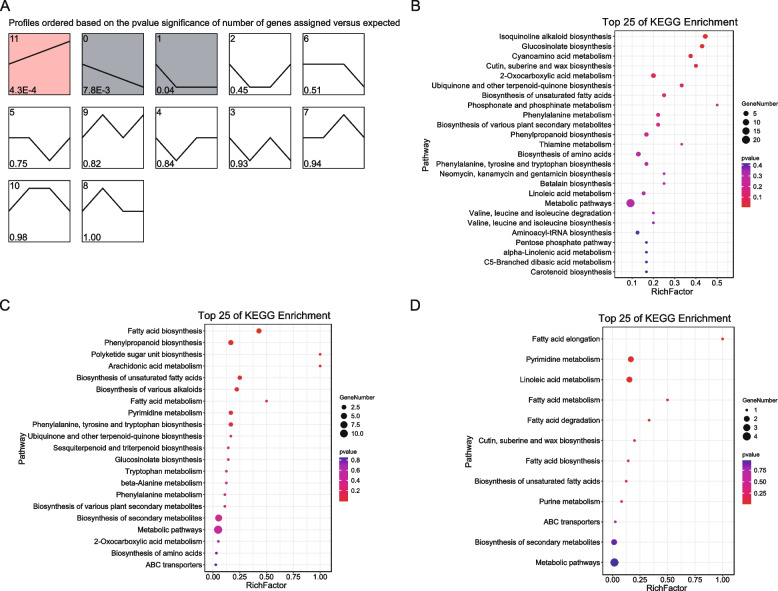
Expression trends of differential metabolites identified in STEM analysis. **A** Metabolite profiles identified in STEM, with profile 11, 0, and 1 highlighted as significant with a specific background color. The numerals in the lower left corner denote the *P-value* of the number of assigned genes versus expected. **B**-**D** KEGG enrichment analysis of metabolites from profile 11 (**B**), profile 0 (**C**), and profile 1 (**D**)

Trend analysis using STEM of the differentially identified metabolites revealed significant enrichment in profiles 11, 0, and 1 (Fig. 5A). KEGG analysis of the metabolites associated with profile 11 indicated enrichment primarily in pathways such as glucosinolate biosynthesis and cyanoamino acid metabolism (Fig. 5B). The metabolites in profile 0 were mainly enriched in pathways including fatty acid biosynthesis, phenylpropanoid biosynthesis, polyketide sugar unit biosynthesis, arachidonic acid metabolism, and biosynthesis of unsaturated fatty acids (Fig. 5C). The metabolites in profile 1 were enriched in pathways such as fatty acid elongation, pyrimidine metabolism, and linoleic acid metabolism (Fig. 5D).

### Weighted gene co-expression network analysis in different drought stress conditions of *Dendrobium*

**Fig. 6:**
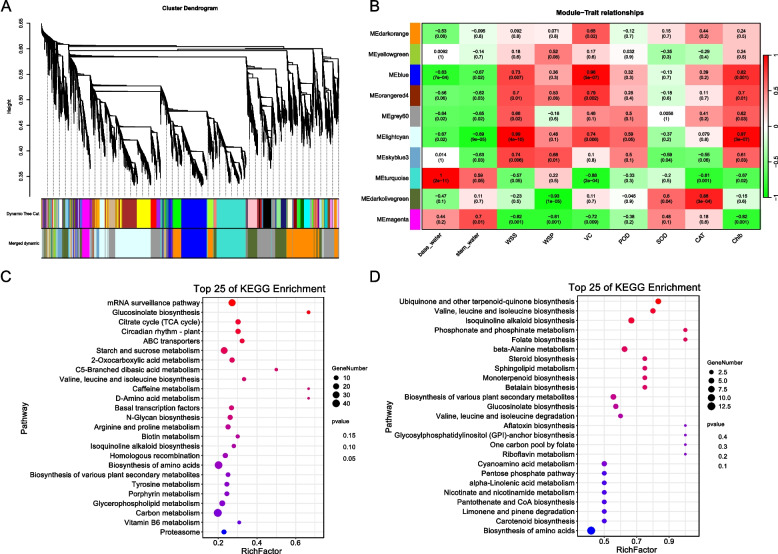
The WGCNA analysis of transcriptomic and metabolome data. **A** The clustering dendrogram of WGCNA, with the original and the merged modules colored. **B** The module-trait relationships evaluated by calculating the correlation of module eigengenes and traits. The correlation coefficients and the corresponding *P-value* are shown. **C**-**D** KEGG enrichment analysis of the common DEGs between profile11 and the light cyan module (**C**) and the common differential metabolites between profile11 and the turquoise module (**D**)

To identify the gene and metabolite modules associated with the physiological properties, WGCNA was conducted using base_water, stem_water, levels of water soluble pectin (WSP), water soluble sugar (WSS), ascorbic acid (VC), superoxide dismutase (SOD), peroxidase (POD), catalase (CAT), and chlorophyll B (ChlB) content as traits. WGCNA partitioned the DEGs into ten distinct modules (Fig. 6A, Table S8). Among these modules, MEblue, MEorangered4, and MElightcyan exhibited significant positive correlations with WSS, with *P-value*s of 0.007, 0.01, and 4e-10, respectively (Fig. 6B, Table S9). Similarly, these modules demonstrated significant positive correlations with VC, with *P-value*s of 5e-07, 0.002, and 0.006, respectively, as well as with Chlb, with *P-value*s of 0.001, 0.01, and 3e-07, respectively (Fig. 6B, Table S9). MEblue negatively correlated with base_water (*P-value* = 7e-04), whereas MElightcyan negatively correlated with stem_water (*P-value* = 9e-05) (Fig. 6B, Table S9). MEskyblue3 expression significantly and positively correlated with WSS (*P-value* = 0.006) and WSP (*P-value* = 0.01) (Fig. 6B, Table S9). MEturquoise positively correlated with base_water (*P-value* = 2e-11) and negatively correlated with VC (*P-value* = 2e-04) and CAT (*P-value* = 0.001) (Fig. 6B, Table S9). MEdarkolivergreen negatively correlated with WSP (*P-value* = 1e-05) and positively correlated with CAT (*P-value* = 3e-04) (Fig. 6B; Table S9). MEmagenta positively correlated with stem_water and negatively correlated with WSS, WSP, VC, and Chlb, with corresponding *P-value*s of 0.001, 0.001, 0.009, and 0.001, respectively (Fig. 6B, Table S9).

The DEGs in significant profile 11, identified by trend analysis, were enriched in the light cyan module (*P-value* < 2.2e-16, Fisher's Exact Test). The common genes between profile11 and the light cyan module were primarily enriched in pathways such as the mRNA surveillance pathway, glucosinolate biosynthesis, citrate cycle (TCA cycle), Circadian rhythm—plant, and ABC transporters (Fig. 6C). Similarly, the differential metabolites in profile 11 identified by trend analysis were enriched in the turquoise module (*P-value* = 0.0079, Fisher's Exact Test). The common genes between profile11 and the turquoise module were mainly enriched in the ubiquinone and other terpenoid-quinone biosynthesis pathways (Fig. 6D).

### Gene regulatory network related to drought tolerance of *Dendrobium*

**Fig. 7:**
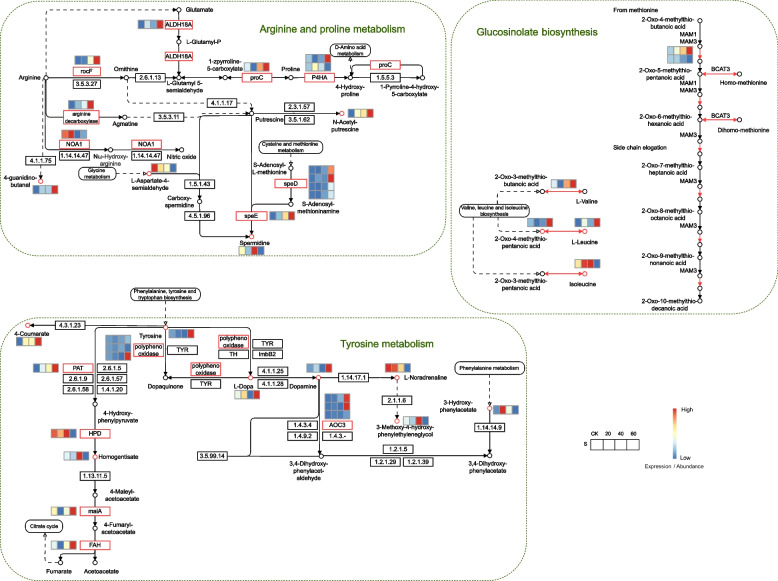
Schematic diagram of drought stress associated pathways, including arginine and proline metabolism, glucosinolate biosynthesis, and tyrosine metabolism, along with heatmaps depicting expression/abundance patterns of DEGs and differentially abundant metabolites. Red boxes and circles indicate the DEGs and metabolites with significant changes under drought stress, respectively

To identify the key genes associated with drought tolerance, we focused on the pathways enriched with DEGs and differential metabolites. Within the arginine and proline metabolic pathways, genes such as *ALDH18A*, *rocF*, *proC*, *P4HA*, arginine decarboxylase, and *speE* showed an increasing trend during drought stress, accompanied by a gradual increase in the metabolites N-acetyl-putrescine and 4-guanidino-butanal, whereas the levels of L-Aspartate-4-semialdehyde declined (Fig. 7). In the glucosinolate biosynthesis pathway, *MAM1* and metabolites such as 2-oxo-3-methylthio-butanoic acid, 2-oxo-4-methylthio-pentanoic acid, and l-leucine, were upregulated (Fig. 7). Genes in the tyrosine metabolism pathway, including polyphenol oxidase, *PAT*, *AOC3*, *maiA*, and *FAH*, along with metabolites 4-coumarate, tyrosine, l-dopamine, and dopamine, showed an increasing trend, whereas l-noradrenaline decreased gradually (Fig. 7).

## Discussion

Water is an essential constituent of plants. Moreover, water is a key environmental factor that influences growth and development. Plants often experience a variety of unfavorable factors throughout their lives; however, drought is at the top of the list of abiotic stressors for plants [14]. To better adapt to drought stress, plants have evolved a variety of complex physicochemical and metabolic mechanisms and strategies to ensure survival and reproduction. When experiencing drought stress, plants will actively accumulate osmoregulatory substances such as soluble sugars, proline, and soluble proteins. To reduce the osmotic pressure inside the cells, plants increase the absorption of water from the soil, reduce the loss of water from the plant, and ensure that the water metabolism of plant cells functions normally. In contrast, drought induces the activation of plant antioxidant enzyme protection systems, which remove harmful substances and reduce damage to plant cell functions [15]. In this study, excessive production of reactive oxygen species (ROS) during plant cell metabolism triggered the oxidation of unsaturated fatty acids (UFAs) under water stress, resulting in the production of large amounts of cytotoxic malondialdehyde (MDA) (Fig. 1B), which disrupted the balance of the plant cell membrane system and disrupted cellular structure and function. Plant antioxidant enzyme systems can convert harmful products such as MDA into low or nontoxic substances through the redox action of superoxide dismutase (SOD), peroxidase (POD), and catalase (CAT) [16]. Therefore, the accumulation of osmoregulatory substances and changes in the activities of key antioxidant enzymes in plant cells are not only an important reflection of the drought adaptive capacity of plants but also of their different drought adaptation strategies.

RNA sequencing has been widely used to study drought resistance mechanisms in *Dendrobium spp*. Transcriptome-wide analysis of *Dendrobium officinale* showed drought stress related microRNAs [17]. miR-159 gene family and their target genes were related with drought stress in *D. officinale* [18]. Researchers conducted an experiment involving the effects of continuous drought treatments on an epiphytic orchid, *D. catenatum*, which was designed to generate 39 mature leaf-tissue RNA-seq datasets with over two billion reads [19]. Whole-transcriptome sequencing revealed the global molecular responses and NAC transcription factors involved in drought stress in *D. catenatum* [20]*.* Three *D. catenatum* species with different photosynthetic pathways were selected for sequencing and transcriptome data analysis after drought treatment [21]. However, the transcriptional regulatory mechanisms underlying drought resistance in *D. nobile* Lindl have rarely been reported. In our study, global transcriptomic changes in different drought stress conditions of *D. nobile* Lindl were detected. Trend analysis revealed that the DEGs were enriched in plant hormone signal transduction and other pathways. Consistent with our results, genome-wide identification and expression analysis have shown that plant hormone signal transduction is crucial for regulating drought stress in *D. chrysotoxum* and *D. catenatum* [22, 23]. Phytohormones play a key role in the adaptation to abiotic stress by influencing growth and development. Plants often activate signaling cascades to survive drought stress, resulting in the accumulation of endogenous hormones and the induction of defense responses [24].

Drought is a common environmental stress with great negative impacts on plant growth, development and geographical distribution as well as agriculture and food production. To identify critical genes associated with drought tolerance in *D. nobile* Lindl., we focused on the arginine and proline metabolism, glucosinolate biosynthesis, and tyrosine metabolism pathways (Fig. 7). Arginine is a nitrogen-rich amino acid with a high N:C ratio (4:6), making it suitable for nitrogen storage [25]. Arginine decarboxylase mainly participates in the arginine metabolism pathway, which was upregulated during drought stress in our results [26]. Catabolism of arginine is the main source of endogenous urea in plants, and recycling of urea is important for plant survival under drought stress conditions [27]. The function of proline, a stress-inducible amino acid, in response to drought stress has been widely elucidated in plants. Its accumulation and metabolism are involved in osmotic adjustment and reactive oxygen species (ROS) scavenging, thus regulating the stability of intracellular structures and membrane systems under drought stress [28]. The proline content is often used as an important index to estimate the severity of stress injury in plants [29]. In this study, maintenance of higher proline metabolism instead of proline accumulation could be one of the main regulatory mechanisms of drought stress in *D. nobile* Lindl., which would be beneficial for the improved glutamate cycle and metabolic homeostasis of amino acids under water-deficient conditions. Glucosinolate, 2-oxo-3-methylthio-butanoic acid, and 2-oxo-4-methylthio-pentanoic acid are secondary metabolites that play fundamental roles in plant resistance to drought stress. These compounds have been found to increase under stress conditions related to the plant adaptive capacity [30]. In our study, the drought-induced accumulation of glucosinolate in *D. nobile* Lindl directly or indirectly controlled stomatal closure, thus preventing water loss [31]. Furthermore, the increase in glucosinolates grown under water stress could be related to the steady water budget and high metabolism of proline, which protects the photosynthetic pigments [32]. The roles of these metabolites in drought tolerance of *D. nobile* will be experimentally validated in future studies.

## Conclusion

In this study, we performed joint transcriptomic and metabolomic analyses to reveal the critical gene regulatory network in response to drought stress in *D. nobile* Lindl. We successfully identified several candidate genes and pathways associated with arginine and proline metabolism, glucosinolate biosynthesis and tyrosine metabolisms, which likely contribute to the regulation of drought stress. In the arginine and proline metabolic pathways, genes such as ALDH18A, rocF, proC, P4HA, arginine decarboxylase, and speE showed an upward trend during drought stress. In the glucosinolate biosynthesis pathway, genes for MAM1 and metabolites such as 2-oxo-3-methylthiobutyric acid, 2-oxo-4-methylthiopentanoic acid and l-leucine were up-regulated. Genes in the tyrosine metabolic pathway, including polyphenol oxidase, PAT, AOC3, maiA, and FAH were up-regulated. The construction of a gene regulatory network in *D. nobile* Lindl will provide further insights into the mechanisms of drought stress response in plants.

## Materials and methods

### Plant material and sampling

The *D. nobile* Lindl. used in the experiment was sourced from the *Dendrobium* planting base in Wanglong Town, Chishui City, Guizhou Province. The plants were cultivated in pots in the experimental greenhouse of Zunyi Normal College with a pot diameter of 24 cm and a height of 26 cm. The potting soil was a uniform mixture of peat, bark, and sphagnum moss at a volume ratio of 1:1:1. The plants were placed on a shade net to avoid direct sunlight exposure.

Potted seedlings of *D. nobile* stems were used as the experimental materials. Four treatment groups were used: CK (control group with adequate water supply), D20 (drought treatment for 20 d), D40 (drought treatment for 40 d), and D60 (drought treatment for 60 d). Different drought treatments were initiated as per the experimental design and all samples were collected simultaneously on the same day.

### Physiological measurement

Physiological measurements included soil substrate moisture content (base_water), stem moisture content (stem_water), and levels of soluble protein (WSP), soluble sugar (WSS), ascorbic acid (VC), superoxide dismutase (SOD), peroxidase (POD), catalase (CAT), and chlorophyll B (ChlB) in *Dendrobium* samples. Base_water and stem_water contents were determined by oven drying. WSP was determined using Coomassie Brilliant Blue staining, whereas WSS was measured using the anthrone colorimetric method. CAT activity was measured using the potassium permanganate titration method, POD activity using the guaiacol method, and SOD activity using the nitro blue tetrazolium (NBT) method. Chlorophyll B was extracted with 90% acetone and measured using a spectrophotometric method [33]. All measurements were normalized to the fresh weights of the samples.

### RNA extraction and RNA-seq analysis

Total RNA was extracted using the cetyltrimethylammonium bromide (CTAB) method, and mRNA molecules with polyA tails were enriched using oligo (dT) magnetic beads. The RNA was then fragmented into approximately 300 bp fragments using ion shearing. First-strand cDNA synthesis was performed using random hexamer primers and reverse transcriptase, followed by second-strand cDNA synthesis using the first-strand cDNA as a template. Subsequent PCR amplification was used for library fragment enrichment, followed by library size selection, targeting fragments of approximately 450 bp. Quality control of the libraries was performed using the Agilent 2100 Bioanalyzer. Next-generation sequencing (NGS) was performed on these libraries using an Illumina sequencing platform and a paired-end (PE) sequencing strategy.

### Transcriptome analysis

Transcriptome sequencing yielded 41,44 M total reads per sample in average. Quality control of sequencing data was performed using FastQC (version 0.11.5) [34] and Cutadapt (version 2.6) [35], with a trimming quality threshold set at Q20. Following quality control, a total of 38.65 M clean reads were obtained. These clean reads were subsequently aligned to the *D. nobile* reference genome (GCA_022539455.1) using HISAT2 software (version 2.1.0, http://ccb.jhu.edu/software/hisat2/index.shtml) [36]. Expression was quantified using HTSeq (version 0.11.0) [37], followed by differential gene expression analysis using DESeq2 (version 1.34.0) [38]. Genes were considered differentially expressed (DEGs) if they exhibited a |log2FoldChange|> 1 and *P* < 0.05. Gene function annotation was performed using Eggnog-mapper (version 2.0.11) [39] and enrichment analysis of Gene Ontology (GO, http://geneontology.org/) terms for DEGs was conducted using topGO (version 2.24.0) [40]. Kyoto Encyclopedia of Genes and Genomes (KEGG, http://www.kegg.jp/) pathway enrichment analysis was performed using hypergeometric testing, and statistical significance was set at *P* < 0.05.

### Sample preparation and LC–MS

The sample preparation protocol involved the following steps. First, an appropriate quantity of sample was weighed accurately and transferred into a 2 mL centrifuge tube [41]. Subsequently, 600 µL of MeOH (stored at −20 °C) containing 2-amino-3-(2-chloro-phenyl)-propionic acid (4 ppm) was added, followed by vortexing for 30 s. Next, 100 mg of glass beads were added to the tube, which was then ground in a tissue grinder for 90 s at 60 Hz. The resulting mixture was then ultrasonicated at room temperature for 15 min. Finally, the sample was centrifuged at 12,000 rpm and 4 °C for 10 min. The supernatant was filtered through a 0.22-μm membrane and transferred into a detection bottle for subsequent analysis using LC–MS detection.

The liquid chromatography (LC) analysis was conducted using an ACQUITY UPLC System (Waters, Milford, MA, USA) equipped with an ACQUITY UPLC ® HSS T3 column (150 × 2.1 mm, 1.8 µm) (Waters, Milford, MA, USA), maintained at 40 ℃. The flow rate and injection volume were set at 0.25 mL/min and 2 μL, respectively. For the LC-electrospray ionization (ESI) ( +)-MS analysis, the mobile phases consisted of (B1) 0.1% formic acid in acetonitrile (v/v) and (A1) 0.1% formic acid in water (v/v). The chromatographic separation followed a gradient elution program: 0–1 min, 2% B1; 1–9 min, 2%–50% B1; 9–12 min, 50%–98% B1; 12–13.5 min, 98% B1; 13.5–14 min, 98%–2% B1; 14–20 min, 2% B1. For LC-ESI (-)-MS analysis, the mobile phases were composed of (B2) acetonitrile and (A2) 5 mM ammonium formate. The chromatographic separation was performed using the following gradient: 0–1 min, 2% B2; 1–9 min, 2%–50% B2; 9–12 min, 50%–98% B2; 12–13.5 min, 98% B2; 13.5–14 min, 98%–2% B2; 14 ~ 17 min, 2% B2 [42].

Mass spectrometric detection of metabolites was performed using a Q Exactive mass spectrometer (Thermo Fisher Scientific, USA) equipped with an ESI source. The instrument operated in Full MS-ddMS2 mode for simultaneous MS1 and MS/MS acquisition, with the following parameters: sheath gas pressure, 30 arb; auxiliary gas flow, 10 arb; spray voltages, 3.50 kV and −2.50 kV for ESI( +) and ESI(-), respectively; capillary temperature, 325 ℃; MS1 scan range, m/z 100–1000; MS1 resolving power, 70,000 FWHM; number of data-dependent MS/MS scans per cycle, 10; MS/MS resolving power, 17,500 FWHM; normalized collision energy, 30 eV; automatic dynamic exclusion time [43].

### Metabolome analysis

The original mass spectrometry raw data files were converted to mzXML format using the MSConvert tool within the Proteowizard software package (v3.0.8789) [44]. Peak detection, filtering, and alignment were performed using the XCMS package to generate a list of quantified compounds [45]. Compounds were identified using public databases, including HMDB [46], LipidMaps [47], and KEGG [48], with parameters set at < 30 ppm.

Data normalization was achieved using the LOESS signal correction method based on QC samples to mitigate systematic errors [49]. Compounds with a relative standard deviation (RSD) > 30% in the QC samples were filtered out during the data quality control. Principal component analysis (PCA), partial least squares discriminant analysis (PLS-DA), and orthogonal partial least squares discriminant analysis (OPLS-DA) were performed using the R package Ropls for dimensionality reduction of the sample data [50]. Permutation tests were conducted to assess model overfitting, and statistical tests were used to calculate the *P-value*. Variable importance in projection (VIP) scores were computed using the OPLS-DA method. Compounds with *P* < 0.05 and VIP > 1 were considered statistically significant metabolites. MetaboAnalyst software was used for functional pathway enrichment and topological analysis of differential metabolites [51].

### Temporal analysis

Temporal analysis of DEGs and differential metabolites was conducted using the Short Time-series Expression Miner (STEM) to identify significant profiles [52]. These profiles were then subjected to KEGG pathway enrichment analysis using the clusterProfiler package, and pathways with *P* < 0.05 were defined as significantly enriched [53].

### Weighted correlation network analysis

Co-expression analysis of DEGs was performed using weighted gene co-expression network analysis (WGCNA) [54]. The correlations between each module and various physiological traits, including base_water, stem_water, WSS, WSP, VC, POD, SOD, CAT, and ChIB, were calculated. Modules with an absolute correlation coefficient > 0.7 and *P* < 0.05 were defined as significantly correlated modules.

### Integrated analyses of transcriptome and metabolome

To align the DEGs and differential metabolite profiles identified by the STEM trend analysis with the modules that correlated significantly with the traits in the WGCNA, we conducted Fisher's Exact Test to assess the enrichment of each profile within each module. Profiles with *P* < 0.05 were considered significantly enriched.

### Statistical analysis

Multiple group comparisons were conducted using Tukey's honest significant difference (HSD) test, with statistical significance defined as *P* < 0.05.

## Supplementary Information

Supplementary Material 1.

## Data Availability

The datasets generated and analysed during the current study are available in the NCBI SRA repository (http://www.ncbi.nlm.nih.gov/bioproject/PRJNA1139333).

## Abbreviations

DEG: Differentially Expressed Gene
DAM: Differentially Abundant Metabolite
KEGG: Kyoto Encyclopedia of Genes and Genomes
PCA: Principal Component Analysis
QC: Quality Control
HSD: Honestly Significant Difference
WGCNA: Weighted Gene Co-expression Network Analysis
STEM: Short Time-series Expression Miner
SOD: Superoxide Dismutase
NBT: Nitroblue Tetrazolium
POD: Peroxidase
CAT: Catalase
ChlB: Chlorophyll B
WSP: Water Soluble Pectin
WSS: Water Soluble Sugar
VC: Ascorbic Acid
